# Pembrolizumab‐induced pancytopenia in a patient with squamous cell lung cancer

**DOI:** 10.1111/1759-7714.13582

**Published:** 2020-08-07

**Authors:** Yuriko Ueki, Manabu Suzuki, Yuriko Horikawa, Hiromu Watanabe, Yoh Yamaguchi, Chie Morita, Akinari Tsukada, Hiroshi Takumida, Yusaku Kusaba, Takashi Katsuno, Yoshie Tsujimoto, Keita Sakamoto, Masao Hashimoto, Junko Terada, Satoru Ishii, Jin Takasaki, Go Naka, Motoyasu Iikura, Shinyu Izumi, Yuichiro Takeda, Masayuki Hojo, Haruhito Sugiyama

**Affiliations:** ^1^ Department of Respiratory Medicine National Centre for Global Health and Medicine Shinjuku‐ku Japan

**Keywords:** Immune checkpoint inhibitor, immune‐related adverse event (irAE), pancytopenia, PD‐L1, squamous cell lung cancer

## Abstract

Immune checkpoint inhibitors (ICIs) are reportedly effective against many kinds of neoplasm, but may be responsible for several kinds of immune‐related adverse events (irAEs). Among these irAEs, the incidence of myelosuppression due to ICIs is relatively low. Corticosteroids are needed to control most cases of myelosuppression. Here, we report an 88‐year‐old woman with squamous cell lung cancer who was administered pembrolizumab. After five cycles of pembrolizumab, she developed severe pancytopenia. The pancytopenia improved under observation without steroid administration after cessation of pembrolizumab. During recovery from this irAE, the patient also maintained long‐term antitumor efficacy.

**Key points:**

**Significant findings of the study:**

There are several kinds of immune‐related adverse events. We encountered a case of pembrolizumab‐induced pancytopenia with squamous cell lung cancer.

**What this study adds:**

Corticosteroids are needed to control most cases of myelosuppression induced by ICIs, but pancytopenia induced by pembrolizumab in our case improved without steroids.

## Graphical abstract

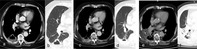

An 88‐year‐old woman with squamous cell lung cancer was administered pembrolizumab. After 5 cycles of pembrolizumab, she developed severe pancytopenia. Pancytopenia improved under observation without steroid administration after cessation of pembrolizumab. During recovery from this irAE, the patient also maintained long‐term anti‐tumor efficacy.

## Introduction

Immune checkpoint inhibitors (ICIs) have ushered in a new era in oncology, significantly improving therapeutic outcomes in cancer patients. Pembrolizumab is an ICI that targets the programmed cell death receptor and activates T cells to fight the cancer. In cases of T cells activated by pembrolizumab, these cells are also likely to attack normal tissues throughout the body. Pembrolizumab administration may thus result in many kinds of immune‐related adverse events (irAEs), including colitis, hepatitis, pneumonitis, endocrinopathies, skin rash, and encephalitis. Some reports have described hematological toxicities due to pembrolizumab, including anemia, thrombocytopenia or leukopenia, but few reports have mentioned multiple‐lineage toxicities. Here, we present a case of pancytopenia‐induced by pembrolizumab which recovered naturally without using steroids.

### Case report

An 88‐year‐old woman presented with episodes of hemoptysis for a week. She was referred to our hospital due to abnormal shadows on chest X‐ray. She had a history of complete surgical resection for ascending colon cancer at 72‐years‐old and basal cell carcinoma of the face at 79‐years‐old, and conservative treatment for early‐stage gastric cancer at 88‐years‐old. She was taking pharmacotherapies for hypertension, angina and dyslipidemia, but had no history of immune‐related diseases. She was an ex‐smoker with three packs/day for 35 years. Neither of her family had multiple cancers except her.

**Figure 1:**
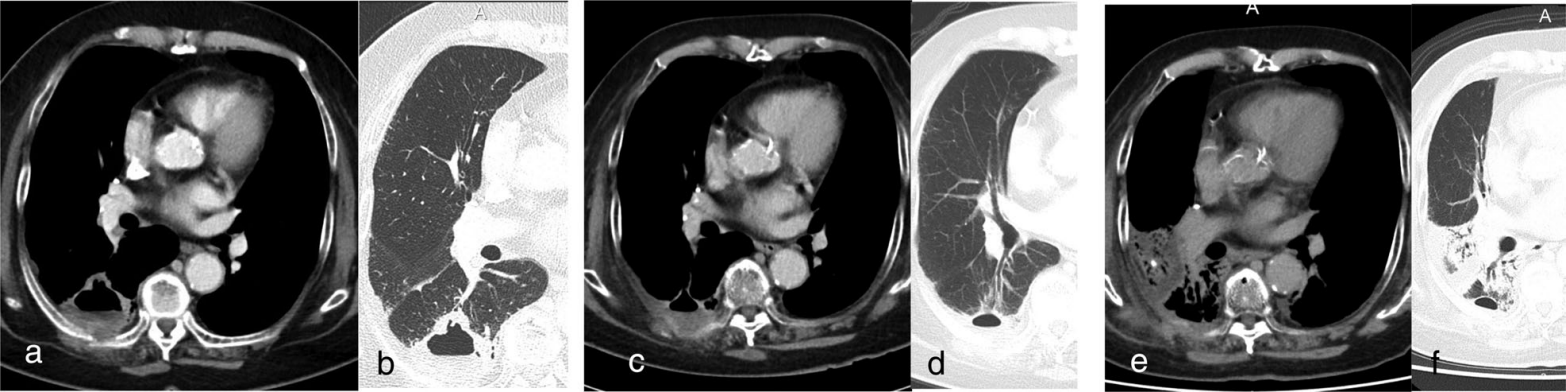
Changes on computed tomography (CT) scan. (**a**, **b**) Before radiation therapy. (**c**, **d**) Radiation pneumonitis observed after radiation therapy. (**e**, **f**) A partial response was evident after four cycles of pembrolizumab. The size of the tumor shrunk from 52 mm to 37 mm after radiation therapy. After four cycles of pembrolizumab the size of the tumor was 28 mm.

**Figure 2:**
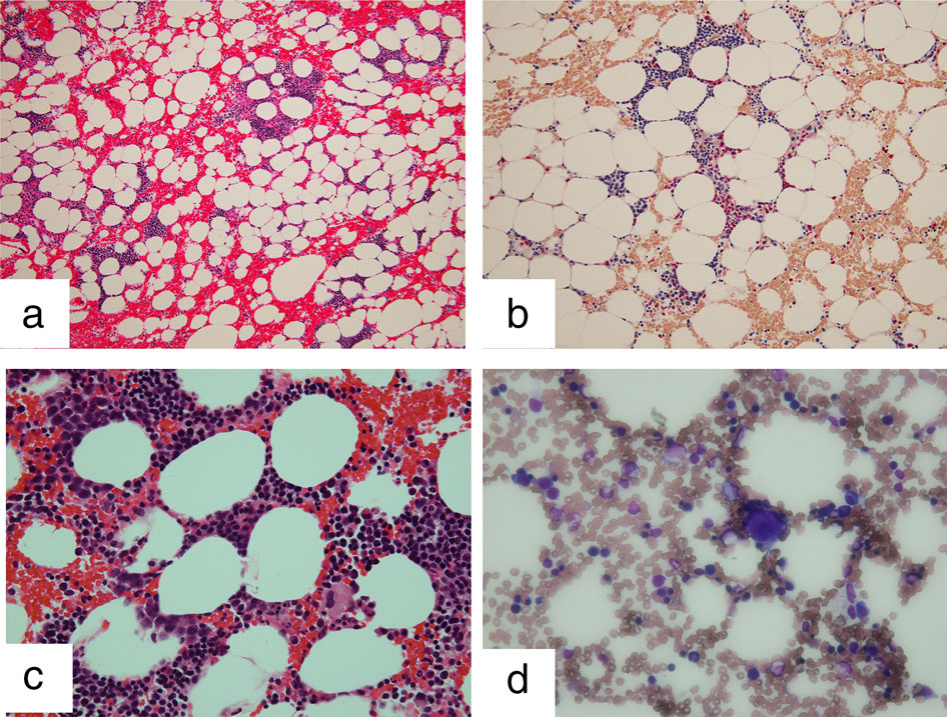
Bone marrow biopsy. (**a**) Hematoxylin and eosin stain, low magnification. (**b**) Giemsa stain, low magnification. (**c**) Hematoxylin and eosin stain, high magnification. (**d**) Giemsa stain, high magnification. No blast proliferation or hemolysis was evident and no significant changes in granulocytes or erythrocytes were observed.

She was diagnosed with squamous cell lung cancer in the right lower lobe, categorized as cT3N1M1a cStage IVA from computed tomography (CT)‐guided biopsy. Tumor proportion score on PD‐L1 was 100% as evaluated by 22C3 antibody. First, radiation therapy (60 Gy/30 fr) was performed because she had recognized back pain because of direct invasion of the cancer around the ribs. She was administered pembrolizumab at 200 mg/bodyweight every four weeks. After four cycles, a partial response (PR) was achieved according to RECIST (Response Evaluation Criteria in Solid Tumors version 1.1) criteria (Fig 1e,f). The tumor maintained PR for a few months after four cycles of pembrolizumab. Because the field of radiation included the primary cancer in the right lower lobe, radiation pneumonitis developed and the primary tumor shrank (Fig 1c,d). After four cycles of pembrolizumab, her pneumonitis was exacerbated but the tumor size shrank further (Fig 1e,f). As a result of radiation therapy and four cycles of pembrolizumab, the tumor shrank without any adverse events. After five cycles of pembrolizumab, routine laboratory investigations showed mild pancytopenia, with a white blood cell count (WBC) of 3.13 × 10^3^/μL. Hemoglobin (Hgb) was 10.8 g/dL and platelet count (Plt) was 5.8 × 10^4^/μL. The sixth cycle of pembrolizumab was suspended after pembrolizumab‐induced pancytopenia was suspected. Severe pancytopenia developed within six months after starting pembrolizumab. At that time, the nadir WBC was 1.92 × 10^3^/μL (grade 3 according to CTCAE version 5), nadir Hgb was 7.8 g/dL (grade 3) and nadir Plt was 2.5 × 10^4^/μL (grade 3). After consultation with a specialist hematologist, the cause of pancytopenia was suspected to be due to pembrolizumab or myelodysplastic syndrome (MDS). Negative results were obtained for antiplatelet antibody. Bone marrow biopsy was performed to confirm the diagnosis, but pathological findings did not indicate any hematological diseases such as MDS (Fig 2). The bone marrow specimen showed normocellular bone marrow. The pancytopenia was considered to have been induced by pembrolizumab. Although oral steroid administration (prednisolone at 1 mg/kg) to manage pancytopenia as a grade 3 irAE was suggested, the patient declined steroids because of the risk of adverse events.

**Figure 3:**
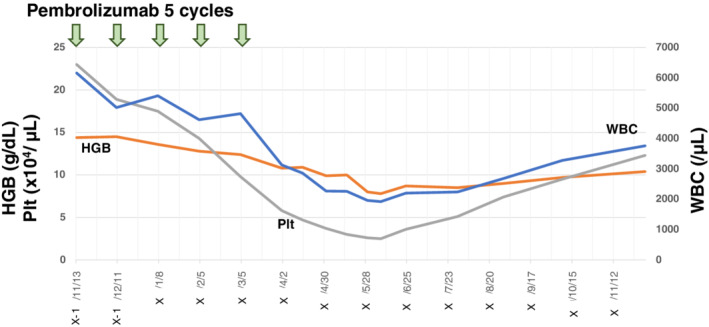
Changes in white blood cells, hemoglobin and platelets. Severe pancytopenia developed after five cycles of pembrolizumab and bone marrow biopsy was performed. Pancytopenia improved naturally with only transfusion of red blood cells six months after ceasing pembrolizumab administration (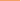) HGB, (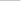) PIt, (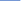)WBC.

The pancytopenia improved naturally within six months after suspension of pembrolizumab, although a red blood cell transfusion was needed to assist patient recovery. No additional severe adverse events were encountered (Fig 3). There was no other agent administered other than pembrolizumab. The antiplatelet antibody and autoimmune antibody related with collagen diseases results were negative. Biopsy of the bone marrow did not show any significant findings. Considering the results and long course of observation, we concluded that pembrolizumab induced pancytopenia by autoimmune reaction. Fortunately, antitumor efficacy continued even after cessation of pembrolizumab and performance status was able to be maintained without any additional treatments for 11 months.

## Discussion

Here, we report a patient with squamous cell lung cancer who developed pancytopenia during treatment with pembrolizumab. We considered the causality of the pancytopenia. Bone marrow biopsy did not indicate any hematological diseases. Severity of the pancytopenia gradually increased with additional pembrolizumab every four weeks. In general, irAE over grade 2 requires interruption of ICIs and the use of corticosteroids.^1^ Steroids are used to suppress excessive immune response by ICIs. Despite clinical practice guidelines, the patient in our case recovered from pancytopenia with observation alone after cessation of pembrolizumab, because she decided against using steroids. As a result, we considered that pancytopenia in this case had been induced by pembrolizumab.

Pembrolizumab can cause many kinds of irAEs. Anemia, neutropenia, thrombocytopenia, leukopenia and lymphopenia are reported to occur in 1%–10%, with febrile neutropenia and eosinophilia occurring in <1%.^2^, ^3^ However, few reports have described pancytopenia as an irAE. The mechanisms by which pembrolizumab induces pancytopenia are unknown. Given the present findings, pembrolizumab‐induced pancytopenia was considered reversible after cessation and washout of the accumulated drug.

We performed a literature search using PubMed in March 2020. We identified 19 reports of hematological adverse events related to pembrolizumab or nivolumab.^8^, ^9^, ^10^, ^11^, ^12^, ^13^, ^14^, ^15^, ^16^, ^17^ Seven reports were related to pembrolizumab.^2^, ^3^, ^4^, ^5^, ^6^, ^7^ Only three reports described pancytopenia, including the present report (Table 1).^2^, ^3^ For nivolumab, 12^8^, ^9^, ^10^, ^11^, ^12^, ^13^, ^14^, ^15^, ^16^, ^17^ reports described hematological adverse events, with only three^17^ showing pancytopenia with lung adenocarcinoma (Table 1). Patients in all the reports were administered steroids or intravenous immunoglobulin, with the exception of one case. There are many reports about treatment with steroids for irAE, but few studies which report the recovery of the condition without steroids. Thus, the case reported here was rare as not only grade 3 pancytopenia occurred, but also the pancytopenia in this patient improved without administration of steroids.

**Table 1:** Hematological adverse events after treatment with pembrolizumab and nivolumab

Author	PD‐1 inhibitor	Disease	Adverse effects	Treatment
Nair *et al*. 2016 ^4^	Pembrolizumab	Metastatic melanoma	AIHA with pure red cell aplasia	Steroids and IVIG
Langer *et al*. 2016^5^	Pembrolizumab	Non‐small cell lung cancer	Anemia, thrombocytopenia, neutropenia	Unknown
Le Roy *et al*. 2016^6^	Pembrolizumab	Metastatic melanoma	Thrombocytopenia	Steroids and IVIG
Atwal *et al*. 2017^3^	Pembrolizumab	Metastatic melanoma	Pancytopenia	Steroids and IVIG
Ogawa *et al*. 2018^7^	Pembrolizumab	Metastatic melanoma	AIHA	Steroids
Ni *et al*. 2019^2^	Pembrolizumab	Metastatic melanoma	AIHA, pancytopenia	Steroids
Ueki *et al*. 2020 (This case)	Pembrolizumab	Non‐small cell lung cancer	Pancytopenia	Nothing
Weber *et al*. 2015^8^	Nivolumab	Metastatic melanoma	Anemia	Steroids
Sharma *et al*. 2016^9^	Nivolumab	Metastatic urothelial melanoma	Anemia, thrombocytopenia	Unknown
Schwab *et al*. 2016^10^	Nivolumab	Squamous cell skin cancer	AIHA	Steroids
Kong *et al*. 2016^11^	Nivolumab	Metastatic melanoma	AIHA	Steroids
Inadomi *et al*. 2016^12^	Nivolumab	Metastatic melanoma	Anemia, thrombocytopenia	Steroids
Palla *et al*. 2016^13^	Nivolumab	Metastatic lung cancer	AIHA	Steroids
Deltombe *et al*. 2017^14^	Nivolumab	Metastatic melanoma	AIHA	Steroids
Khan *et al*. 2017^15^	Nivolumab	Metastatic melanoma	AIHA	Steroids
Yuki *et al*. 2017^16^	Nivolumab	Cardiac metastatic melanoma	Pure red cell aplasia	Steroids
Michot *et al*. 2017^17^	Nivolumab	Lung adenocarcinoma	Pancytopenia, AIHA	IVIG
Michot *et al*. 2017^17^	Nivolumab	Lung adenocarcinoma	Pancytopenia, AIHA	IVIG, GCF
Michot *et al*. 2017^17^	Nivolumab	Lung adenocarcinoma	Pancytopenia, AIHA	Steroids, GCF

AIHA, autoimmune hemolytic anemia; IVIG, intravenous immunoglobulin.

Steroids may cause various side effects, which sometimes threaten a patient's condition. Moreover, it is possible that steroids decrease the antitumor effects of treatment. However if we can manage irAE without steroids, patients should not be concerned about the possible side effects of steroids and the anti‐tumor effects. In the case reported here, pembrolizumab achieved a durable tumor response and transient pancytopenia, and the tumor size decreased after improvement of the pancytopenia. Osa *et al*. reported nivolumab binding of T cells after discontinuation of more than 20 weeks and a possibility of residual efficacy of nivolumab after cessation.^18^ Pembrolizumab is one of ICIs as nivolumab, so we thought that pembrolizumab might also have a durable tumor response. According to some reports, a combination of ICIs and radiation therapy have achieved high rates of local control.^19^, ^20^ The case reported here was treated with pembrolizumab after radiation therapy, and a similar anti‐tumor effect might be achieved.

In conclusion, pembrolizumab may induce pancytopenia in some patients and the pancytopenia may improve without steroids as a reversible irAE. In daily clinical practice, close attention should be paid to hematological data during ICI treatment.

## Disclosure

The authors state that they have no conflicts of interest (COI).

YT has received grants from Boehringer Ingelheim and Chugai Pharmaceutical outside of this study.
